# Supplementary material for: An agent-based model of cattle grazing toxic Geyer's larkspur
Source: PLoS One. 2018 Mar 22;13(3):e0194450. doi: 10.1371/journal.pone.0194450 (PMC5864015; doi:10.1371/journal.pone.0194450)
Supplement: S1 Code — (HTML) [file pone.0194450.s001.html]

```
:Copyright 2017 Kevin Jablonski
;Please contact kevin.jablonski@colostate.edu with all inquiries

Extensions [GIS]
Globals [herd-cohesion site-radius herd-up-count vegdist larkdist site-tolerance water-locations waterers cow-count forage-concentration watering site-change site-change-targets site-change-count
          mean-times-grazed day-count mean-consumption-level death-count tolerance-death attraction-death death-leader death-follower death-independent danger-zone tolerance-danger attraction-danger]
patches-own [SAVI water-status forage-mass n-forage-mass MSAL-content cluster-center fence gate times-grazed]
cows-own [MSAL-tolerance larkspur-attraction consumption-level MSAL-level total-MSAL-intake daily-MSAL-intake max-daily-MSAL-intake role herdmates mean-herd-distance water ready-to-go distance-traveled]
breed [cows cow]


;************
;  SETUP- uses real landscape for pasture 16 of Maxwell ranch
;************

to setup
  clear-all
   resize-world 0 1662 0 1579 ; set to size of Maxwell pasture 16 in square meters (plus borders)
   set-patch-size 1
   setup-pasture-GIS
   setup-waterers-GIS
   setup-larkspur-GIS
   setup-slope-GIS
   ;set-default-shape cows "cow"
   let initialx random 1663
   let initialy random 1580
   create-cows AU-per-hectare * (count patches with [forage-mass > 0] / 10000) * 0.909 ;sets number of cows to match AU-per hectare- each cow 1.1 AU
    [
     set cow-count count cows
     set size 3
     set MSAL-tolerance random-normal 4000 333.333 ;continuous normal distribution with 8mg/kg as mean, range ~+/- 25% (3000-5000 mg; 6-10 mg/kg)
     set larkspur-attraction random-normal 1 0.0833 ;continuous normal distribution with 1 as mean, range +/- 25% (0.75-1.25)
     setxy initialx initialy ;arbitrary spot, can be gate if needed
     set water 0 ; start by finding water
    ]
   setup-roles
   file-open "daily-MSAL-output.txt"
   ;file-type "Model run"
   ;file-type AU-per-hectare
   ;file-type " "
   ;file-show herd-cohesion-factor
   reset-ticks
end

;*******************
; SETUP PASTURE GIS
;******************

to setup-pasture-GIS
  set vegdist gis:load-dataset "SAVI.asc"  ; load GIS SAVI layer
  gis:set-world-envelope-ds gis:envelope-of vegdist
  ask patches
  [
    set SAVI gis:raster-sample vegdist self ;set SAVI value for each patch (range=0-99)
    set forage-concentration kgs-per-hectare / 10 ;take input as kgs/ha, convert to g/1.0 m2 (patch)
    ifelse (SAVI >= 0) ;exclude outside values
    [
      set forage-mass forage-concentration * (0.3043 * 2.7183 ^ (0.0712 * SAVI)); nonlinear exponential formula for lowest value being 1/3 of mean and highest being 3 times mean
      set n-forage-mass mean [forage-mass] of patches in-radius 3
      set pcolor scale-color green forage-mass 200 -20
      if forage-mass = 0 [set pcolor brown]
    ]
    [
      set pcolor black
      set fence 1
      set SAVI -1
      set times-grazed 1
    ] ;sets outside pasture as black and identies it as fence and grazed so it is not targeted
  ]
end

;********************
; SETUP WATERERS GIS
;********************

to setup-waterers-GIS
  set water-locations gis:load-dataset "water.asc" ; load GIS water layer
  ask patches
  [
    set water-status gis:raster-sample water-locations self ; set water status for patch
  ]
  set waterers patch-set ( patches with [water-status = 1] )
  ask waterers
    [set pcolor blue set forage-mass 0]

end


;********************
; SETUP LARKSPUR GIS
;********************

to setup-larkspur-GIS
  if larkspur-on?
  [
    set larkdist gis:load-dataset "larkspur.asc"  ; load GIS larkspur layer
    ask patches
    [
      ifelse gis:raster-sample larkdist self > 0
        [
          if SAVI > 20
            [
              set MSAL-content gis:raster-sample larkdist self * MSAL-concentration * mean-larkspur-mass * 1.5 ; in areas of high SAVI, set larkspur mass to 50% higher than median- numbers based on data from Maxwell
              set pcolor scale-color violet MSAL-content 200 -40
            ]
          if SAVI > 10 and SAVI <= 20
           [
              set MSAL-content gis:raster-sample larkdist self * MSAL-concentration * mean-larkspur-mass ; in areas of moderate SAVI, set larkspur mass to median
              set pcolor scale-color violet MSAL-content 200 -40
           ]
          if SAVI <= 10
          [
              set MSAL-content gis:raster-sample larkdist self * MSAL-concentration * mean-larkspur-mass * .5 ; in areas of low SAVI, set larkspur mass to 50% lower than median
              set pcolor scale-color violet MSAL-content 200 -40
            ]

        ]
        [set MSAL-content 0]
    ]
  ]
end

;********************
; SETUP SLOPE GIS
;********************
to setup-slope-GIS ;don't think that slope is necessary to larkspur question in pasture 16

end


;***************
;  SETUP ROLES
;***************
to setup-roles
  ask min-n-of round (.05 * cow-count) cows [who]  ; 5% of cows are leaders
    [set role 1 set color white]
  ask max-n-of round (0.1 * cow-count) cows [who]  ; 10% are independents
    [set role 3 set color orange]
  ask cows
    [
      if role = 0                              ;rest are followers
        [set role 2 set color black]
    ]
  set site-tolerance round (4 * ln AU-per-hectare + 10) ; sets tolerance for site change in leaders- formula derived from trial and error
end


;*************
;     GO
;*************
to go
 set herd-cohesion herd-cohesion-factor * 10 ; herd-cohesion-factor x 10 to make herding range 10-100m
 set site-radius (sqrt((110 * herd-cohesion-factor * cow-count - 90) / pi)); formula sets radius so area per cow on new site to range from 20 m2 to 1010 m2 depending on cohesion factor
 ask cows
   [
    if patch-ahead 1 = nobody or [fence] of patch-ahead 1 = 1 ; if on edge of world or edge of GIS layer
       [set heading heading - 180]
   ]
 set watering 0 ;this and next are 0 if watering or site change not occurring, so reset to 0 here
 set site-change 0
 check-water
 check-site-change
 assess-herd
 assess-larkspur-tox
 if video-on? [export-view (word "E://Google Drive/CSU-PhD/ABM/Videos/raw/" (10000 + ticks) ".png")]
 if remainder ticks 100 = 0 ;check following only every 100 ticks
   [
      set mean-times-grazed mean [times-grazed] of patches
      ;if ((mean [consumption-level] of cows / 12500) * cow-count * 1.1) / 30 > 150 [stop] ; allows for setting AUM limit
      ask waterers [set pcolor blue]
   ]
  set mean-consumption-level mean [consumption-level] of cows
  tick
end

;*************
;   EAT
;************
to eat
    if times-grazed = 0 ;cows will graze a bit differently depending on whether patch has been grazed before
        [
        set consumption-level consumption-level + 0.4 * forage-mass ;consume 40% of available forage in patch
        set water water - (0.4 * forage-mass)
        set forage-mass forage-mass - (0.4 * forage-mass)
        set total-MSAL-intake total-MSAL-intake + (0.4 * MSAL-content * larkspur-attraction)
        set daily-MSAL-intake daily-MSAL-intake + (0.4 * MSAL-content * larkspur-attraction)
        set MSAL-level MSAL-level + (0.4 * MSAL-content * larkspur-attraction)
        set MSAL-content MSAL-content - (0.4 * MSAL-content * larkspur-attraction)
        ]
    if times-grazed = 1
        [
        set consumption-level consumption-level + 0.5 * forage-mass
        set water water - (0.5 * forage-mass)
        set forage-mass forage-mass - (0.5 * forage-mass)
        set total-MSAL-intake total-MSAL-intake + (0.5 * MSAL-content * larkspur-attraction)
        set daily-MSAL-intake daily-MSAL-intake + (0.5 * MSAL-content * larkspur-attraction)
        set MSAL-level MSAL-level + (0.5 * MSAL-content * larkspur-attraction)
        set MSAL-content MSAL-content - (0.5 * MSAL-content * larkspur-attraction)
        ]
    if times-grazed > 1
        [
        set consumption-level consumption-level + 0.6 * forage-mass
        set water water - (0.6 * forage-mass)
        set forage-mass forage-mass - (0.6 * forage-mass)
        set total-MSAL-intake total-MSAL-intake + (0.6 * MSAL-content * larkspur-attraction)
        set daily-MSAL-intake daily-MSAL-intake + (0.6 * MSAL-content * larkspur-attraction)
        set MSAL-level MSAL-level + (0.6 * MSAL-content * larkspur-attraction)
        set MSAL-content MSAL-content - (0.6 * MSAL-content * larkspur-attraction)
        ]

   set pcolor scale-color green forage-mass 140 0
   if MSAL-content > 0
    [set pcolor scale-color violet MSAL-content 200 -40]
   set times-grazed times-grazed + 1
   if times-grazed = 2 [set pcolor yellow]
   if times-grazed > 2 [set pcolor red]
end

;******************
; CHECK WATER
;*****************
to check-water
  ask cows with [role = 1] ;only leaders make water decision
   [
      if water <= 0
       [
          set watering 1 ; global variable tells rest of cows to go to water, and ensures that only go-to-water happens in tick when it is needed
          ask cows [go-to-water]
      ]
   ]
end

;*****************
;  GO TO WATER
;*****************
to go-to-water
  let target min-one-of waterers with [count cows-here < 3] [distance myself] ;distribute cows over available waterering spaces
  set distance-traveled distance-traveled + distance target
  move-to target
  set water 6250
 if role = 1
  [set ready-to-go site-tolerance - 1] ;after watering, lead cows are intolerant of poor grazing around water and will leave quickly if overgrazed
end

;********************
; CHECK SITE CHANGE
;********************
to check-site-change
 if watering = 0 ;ensures that site change doesn't happen in same tick as go to water
 [
    ask cows with [role = 1] ;only leader cows make site-change decision
      [
        if mean [times-grazed] of patches in-radius 10 > (0.5 * mean-times-grazed + 1.2) ; makes them want to leave sites that have been grazed moderately to heavily, relative to rest of pasture- formula advances threshold slowly
          [set ready-to-go ready-to-go + 1]
        if ready-to-go >= site-tolerance
        [
          ifelse max [water] of cows with [role = 1] > 500 ;if getting thirsty, goes to water instead of new site
           [
              ask cows with [role = 1] [set ready-to-go 0]
              set site-change 1 ;global variable ensures that site change doesn't happen in same tick as assess-herd / environ movement
              let target-patches max-n-of 5 patches in-radius 1000 with [mean [times-grazed] of patches in-radius 5 < .75 * mean-times-grazed] [n-forage-mass] ; gives lead cows "vision" of 1km for choosing new, relatively ungrazed, site
              let target-patch min-one-of target-patches [distance myself]
              ifelse target-patch != nobody
                [
                 ask target-patch
                 [
                  set site-change-targets patches in-radius (site-radius) with [times-grazed < mean-times-grazed * 2]] ;creates new site
                  set site-change-targets max-n-of (cow-count * 10) site-change-targets [forage-mass] ;cuts down number of available site-change targets to best available
                  ask cows with [role = 1] [change-site] ;new site apportioned according to role
                  ask cows with [role = 2] [change-site]
                  ask cows with [role = 3] [change-site]
                  set site-change-targets no-patches
                  set site-change-count site-change-count + 1
                ]
                [
                  print "no target patch" ; if there is no patch meeting site-change requirements- should be very rare at all but heaviest usage levels
                  ask max-one-of patches in-radius 1000 [forage-mass]
                   [set site-change-targets patches in-radius (0.5 * herd-cohesion)]
                  ask cows [change-site]
                  set site-change-targets no-patches
                  set site-change-count site-change-count + 1
                ]

          ]
          [
             set watering 1 ; if done with current site but nearly in need of water, go to water instead
             ask cows [go-to-water]
          ]
        ]
       ]
  ]
end

;****************
; CHANGE SITE
;****************
to change-site
  let target max-one-of site-change-targets with [not any? cows-here] with [not any? cows-on neighbors4] [forage-mass] ;go to best empty site-change-target, neighbors4 gives them reasonable space
  set distance-traveled distance-traveled + distance target
  face target
  move-to target
  eat
end

;****************
;   ASSESS HERD
;****************
to assess-herd
 if watering = 0 and site-change = 0 ;ensures that assess-herd/environ movement don't happen if either go-to-water or site-change have happened
  [
  ask cows
  [
    if role < 3 ;leaders and followers
     [
      set herdmates min-n-of 20 cows [distance myself] ;use nearest 20 cows, subgroups
      set mean-herd-distance mean [distance myself] of herdmates
      ifelse mean-herd-distance > herd-cohesion
        [
          set herd-up-count herd-up-count + 1
          let x-centroid mean [xcor] of herdmates
          let y-centroid mean [ycor] of herdmates
          facexy x-centroid y-centroid
          let empty-patches patches in-cone 25 45 with [not any? cows-here] with [distance myself > 10] ; choose patch in direction of herd center
          if any? empty-patches
           [
              let target max-one-of empty-patches [forage-mass]
              set distance-traveled distance-traveled + distance target
              face target
              move-to target
              eat
          ]
        ]
        [environmental-movement]
     ]
  if role = 3 ; independent cows less herd-dependent and also pushed out of center of herd
   [
    set herdmates min-n-of 20 cows [distance myself]
    set mean-herd-distance mean [distance myself] of herdmates
    ifelse mean-herd-distance > (2.5 * herd-cohesion) or mean-herd-distance < (0.5 * herd-cohesion)
     [
       let x-centroid mean [xcor] of herdmates
       let y-centroid mean [ycor] of herdmates
       if mean-herd-distance > (2.5 * herd-cohesion) ;2.5 is a guesstimate
        [
          facexy x-centroid y-centroid
          let empty-patches patches in-cone 25 45 with [not any? cows-here] with [distance myself > 10]
          if any? empty-patches
            [
              let target max-one-of empty-patches [forage-mass]
              set distance-traveled distance-traveled + distance target
              face target
              move-to target
              eat
            ]
          ]
        if mean-herd-distance < (0.5 * herd-cohesion) ;also a guesstimate
          [
            facexy x-centroid y-centroid
            set heading heading - 180
            let empty-patches patches in-cone 25 45 with [not any? cows-here] with [distance myself > 10] with [fence = 0]
            ifelse any? empty-patches
              [
               let target max-one-of empty-patches [forage-mass]
               set distance-traveled distance-traveled + distance target
               face target
               move-to target
               eat
              ]
              [
                let target one-of patches in-radius 25 with [fence = 0] with [not any? cows-here] with [distance myself >= 10] with [distance myself <= 25]
                set distance-traveled distance-traveled + distance target
                face target
                move-to target
              ]
          ]
     ]
      [environmental-movement]
  ]
 ]
]
end

;*************************
;  ENVIRONMENTAL MOVEMENT
;*************************
to environmental-movement
     if mean [times-grazed] of patches in-radius 10 < 0.5 ;if in an ungrazed area
        [
        let empty-patches patches in-cone 2 90 with [not any? cows-here]
          if any? empty-patches
            [
              let target max-one-of empty-patches [forage-mass]
              set distance-traveled distance-traveled + distance target
              face target
              move-to target
              eat
            ]
        ]
     if mean [times-grazed] of patches in-radius 10 >= 0.5 ;if in moderately well grazed area
       [
      let empty-patches patches in-cone 10 90 with [not any? cows-here] with [times-grazed = 0]
          ifelse any? empty-patches ;makes an ungrazed patch the preference, but not a requirement
            [
              let target max-one-of empty-patches [forage-mass]
              set distance-traveled distance-traveled + distance target
              face target
              move-to target
              eat
            ]
           [
             let empty-patches-b patches in-cone 10 90 with [not any? cows-here]
             if any? empty-patches-b
                  [
                   let target max-one-of empty-patches-b [forage-mass]
                   set distance-traveled distance-traveled + distance target
                   face target
                   move-to target
                   eat
                  ]
         ]
       ]
end

;**********************
; ASSESS LARKSPUR TOX
;*********************
to assess-larkspur-tox ; enables analysis of larkspur consumption
  if mean-consumption-level / 12500 >= (day-count + 1)
  [
    set day-count day-count + 1
    ask cows
      [
          if daily-MSAL-intake > max-daily-MSAL-intake
              [set max-daily-MSAL-intake daily-MSAL-intake]
          if MSAL-level >= MSAL-tolerance
              [
                set death-count death-count + 1
                set tolerance-death tolerance-death + MSAL-tolerance
                set attraction-death attraction-death + larkspur-attraction
                if role = 1 [set death-leader death-leader + 1]
                if role = 2 [set death-follower death-follower + 1]
                if role = 3 [set death-independent death-independent + 1]
                set MSAL-level 0
               ]

          if MSAL-level >= 0.5 * MSAL-tolerance and MSAL-level < MSAL-tolerance
            [
              set danger-zone danger-zone + 1
              set tolerance-danger tolerance-danger + MSAL-tolerance
              set attraction-danger attraction-danger + larkspur-attraction
            ]
          file-type behaviorspace-run-number file-type "-" file-type herd-cohesion-factor file-type "-" file-type AU-per-hectare file-type "-" file-print precision daily-MSAL-intake 3
          set daily-MSAL-intake 0
          set MSAL-level MSAL-level * 0.5

     ]
  ]
end
```
